# Factors That Affect Survival Outcomes in Patients with Endometrial Clear Cell Carcinoma

**DOI:** 10.3390/jcm11236931

**Published:** 2022-11-24

**Authors:** Vasilios Pergialiotis, Dimitrios Haidopoulos, Theano Christodoulou, Ioannis Rodolakis, Ioannis Prokopakis, Michalis Liontos, Alexandros Rodolakis, Nikolaos Thomakos

**Affiliations:** 1 First Department of Obstetrics and Gynecology, Division of Gynecologic Oncology, “Alexandra” General Hospital, 11528 Athens, Greece; 2 First Department of Propedeutic Surgery, National Kapodistrian University of Athens, Hippocration Hospital, 11528 Athens, Greece

**Keywords:** clear cell carcinoma, endometrial cancer, survival, recurrence

## Abstract

Background: Clear cell carcinoma (CCC) is a rare aggressive histologic subtype of endometrial cancer with a high relapse rate. In the present study, we sought to evaluate the prognostic factors of disease relapse and overall survival. Methods: We conducted retrospective cohort studies that included endometrial CCC patients treated at our institution. Predictive variables of survival outcomes were evaluated considering factors that determine the survival of patients with endometrioid carcinoma. Results: Fifty-five patients with a median age of 68 years and a median follow-up period of 31 months were included in the present study. Recurrence-free and overall survival rates did not differ among patients with early-stage and advanced-stage disease (RFS HR 1.51 (95% CI 0.63, 3.61), OS HR 1.36 (0.56, 3.31)). Patients with upper abdominal metastases had significantly shorter progression-free and overall survival intervals (*log-rank* < 0.001). The Gehan-Breslow-Wilcoxon analysis indicated worse survival rates for patients with advanced disease (*p* = 0.040); however, the *log-rank* test that gave equal weight to all time points did not reveal significant differences (*log-rank* = 0.576). Conclusion: Clear cell carcinoma is an aggressive histologic subtype of endometrial cancer that seems to be moderately affected by known predictors of survival rates in endometrioid carcinoma patients, except for the disease stage. Further research is needed to evaluate whether the molecular profiling of these patients may help predict survival outcomes.

## 1. Introduction

Uterine cancer is the most common gynecologic malignancy, accounting for more than 400,000 cases in a yearly worldwide setting [1]. More than 90% of cases arise in endometrial cancer, with the endometrioid subtype being the most common type of endometrial carcinoma, which is encountered in 75–80% of cases [2]. Rarer histologic subtypes included serous carcinoma (10%), carcinosarcoma and undifferentiated carcinoma (5% each) and clear cell carcinoma (CCC) (2–5%). Molecular taxonomy of endometrial carcinomas subgroups cases in two major subclasses, Type I, which are less aggressive and hormone-dependent and Type II, which are more aggressive and less hormone-dependent [3,4]. Non-endometrioid endometrial cancer is classified into Type II carcinomas, accounting for approximately 40% of cases and is associated with inferior progression-free and overall survival rates [2].

Survival rates and factors that affect survival parameters in cases with CCC have not been elucidated and a recent expert panel report suggests that this type is poorly understood [5]. As in other forms of endometrial cancer, surgery is the cornerstone of treatment for CCC carcinoma. In advanced stages, optimal tumor reduction should be ensured, including the removal of bulky lymph nodes, whereas in early-stage cases, complete lymphadenectomy should be performed, which usually involves pelvic and paraortic lymph nodes [6,7]. The importance of adjuvant therapy in early-stage disease is a subject of debate; however, both the National Comprehensive Cancer Network^®^ (NCCN^®^) and European Society of Gynecologic Oncology (ESGO) guidelines suggest that CCC cases should be offered adjuvant treatment [2,8]. In the absence of myometrial invasion, patients may be treated with vaginal brachytherapy alone, whereas cases with myometrial invasion irrespective of the stage of the disease may be offered chemotherapy and radiotherapy or chemotherapy alone [5]. In recent years, with the adoption of molecular taxonomy methods, immune checkpoint inhibitors have been instituted in the treatment plan for advanced and recurrent endometrial cancer, including cases with CCC.

Overall survival rates of patients with CCC fall within the range of 40–50 months in the international literature, with recurrences occurring within 2 years from diagnosis, thus denoting the aggressiveness of this histologic subtype, which is considered worse compared to grade endometrioid endometrial carcinomas [9,10,11]. Factors that affect survival include stage of the disease, depth of myometrial invasion, lymphovascular space involvement (LVSI) and use of adjuvant therapy (chemotherapy and radiotherapy). In the present retrospective study, we describe survival rates of CCC cases treated in our center, as well as factors associated with survival.

## 2. Materials and Methods

### 2.1. Study Design

The study was based on a retrospective chart review of the records of all patients diagnosed with clear cell carcinoma in our institution between 2010 and 2018. Mixed types of epithelial carcinoma, as well as patients with carcinosarcoma, were excluded from inclusion. All patients were primarily surgically treated, mainly with total hysterectomy and bilateral salpingo-oophorectomy, bilateral pelvic lymphadenectomy with or without paraortic lymphadenectomy. Omentectomy was reserved for cases with apparent disease, as well as for cases with enlarged lymph nodes. The present study is in accordance with the Declaration of Helsinki concerning animal and human rights and was approved by the institutional review board of our hospital prior to its onset (IRB approval number: 275/2022).

Staging was based on the latest FIGO (International Federation of Gynecology and Obstetrics) classification for endometrial cancer [2]. Cases assigned in a FIGO stage prior to the publication of these latest guidelines were re-evaluated by a senior pathologist. Early-stage disease was predefined as stage I and II CCC and advanced stage as stages III and IV.

Adjuvant chemotherapy was provided in all cases using a platinum-based scheme, mainly carboplatin and paclitaxel. Cases with cervical disease were also offered brachytherapy and those with disease extending in pelvic structures (parametria, lymph nodes, etc.) were offered external beam radiotherapy. Molecular taxonomy is currently strongly considered in decision making for adjuvant therapy, even for CCC cases; however, in our institution it has been instituted that last 2 years; hence, information referring to MSI (micro-satellite instability), p53- and POLE- mutations were not available in the present dataset.

### 2.2. Outcomes

Overall survival and progression-free survival were the primary outcomes that were assessed in our study in terms of absolute differences and hazard ratios. Progression-free survival was defined as the interval between the initial surgical treatment of the patient and the date of recurrence. In cases with sub-optimal tumor excision, this was evaluated only if adjuvant treatment resulted in no evidence of disease in the follow-up evaluation with routine computed tomography or magnetic resonance imaging scans. Overall survival was defined as the interval between the initial surgical treatment of the patient and the date of death. In our study, all cases died from cancer-related causes; hence, overall survival was equal in terms of cancer-specific survival. Selected factors that could potentially determine survival outcomes were also evaluated, including patient age and body mass index (BMI), FIGO stage, maximum intrauterine tumor diameter, presence of lymphovascular invasion (LVSI), upper abdominal surgery (including omentectomy), use of adjuvant chemotherapy and adjuvant radiotherapy, as well as presence of residual tumor following the primary operation. 

Post-recurrence survival was also evaluated and a comparison among cases that received chemotherapy following ascertainment of recurrence and those that did not was performed. Recurrence-free survival was defined as the interval between the first recurrence of the disease and the date of patient death.

### 2.3. Statistical Analysis

Statistical analysis was performed using the SPSS 20.0 program (IBM Corp. Released 2011. IBM SPSS Statistics for Windows, Version 20.0. IBM Corp: Armonk, NY, USA). Evaluation of the normality of distributions was performed with graphical methods and the Kolmogorov-Smirnoff analysis. Cox regression analysis (Enter method) was carried out in order to assess the independent effect of the stage of disease, maximum tumor diameter, presence of LVSI, use of adjuvant chemotherapy, use of adjuvant chemotherapy and presence of residual disease at the end of the operation on survival outcomes (PFS and OS). The impact of chemotherapy on post-recurrence survival was also evaluated. The Kaplan–Meier method was carried out to obtain a graphical representation of survival analyses. The level of significance for all analyses was set to *p* < 0.05.

## 3. Results

Overall, 55 patients were included in the present retrospective cohort. Their median age was 68 years (60–76), and the median follow-up period was 31 months (6–72). The largest proportion of the patients were classified at FIGO stage III due to lymph node metastases, followed by FIGO stage I (Table 1). The presence of LVSI was observed in 39 cases (71%) and upper abdominal surgery (excluding omentectomy) was required in 9 cases. The majority of cases received post-operative chemotherapy (90%) and radiotherapy was offered in 64% of cases. Following completion of the primary operation, residual tumors were encountered in 13% of cases.

Twenty-seven patients recurred during the follow-up period accounting for approximately half of the entire cohort and 22 died from the disease (40%). The progression-free survival of the entire cohort was 35.77 months (95% CI 29.10, 42.45). The overall survival reached 47.35 months (95% CI 39.89, 54.82). The 3-year relapse rates and overall survival rates were 36.8% and 42%, respectively, whereas the 5-year relapse rates and overall survival rates were 50.3% and 60%, respectively.

**Figure 1:**
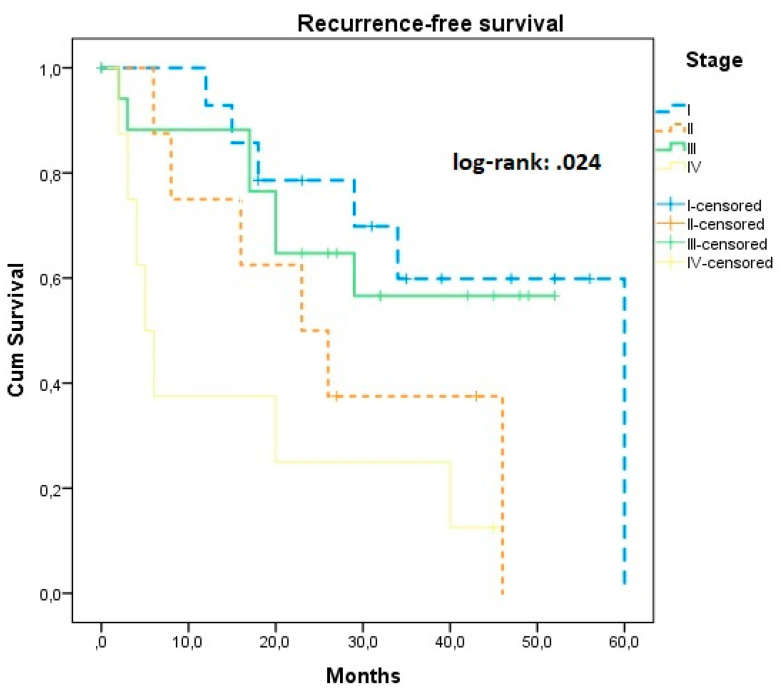
Kaplan–Meier plot of recurrence-free survival according to the stage of the disease.

Survival analysis per investigated factor that could influence survival rates is presented in Table 2. Briefly, we observed that stage was directly associated with progression-free survival (*log-rank* = 0.024), a finding that was mainly influenced by stage IV patients (*log-rank* = 0.007) (Figure 1). A progressive decline in overall survival intervals was also observed, a finding that was marginally significant (*log-rank* = 0.048) and primarily attributed to stage IV patients. This latter group also had a considerably higher risk of disease relapse, as well as death.

Separating cases with early-disease from those with advanced stage, we observed that the recurrence-free survival was shorter but non-statistically significant in patients with advanced-stage disease (30.91 (22.71, 39.13) vs. 37.10 months (27.70, 46.50), *log-rank* = 0.722). Similar results were obtained for overall survival (44.99 (34.20, 55.78) vs. 50.99 months (40.27, 61.72, *log-rank* = 0.490). Hazard ratios of recurrences and survival rates also did not reveal significant differences (PFS HR 1.51 (95% CI 0.63, 3.61) and OS HR 1.36 (0.56, 3.31)). Neither the presence of LVSI nor the use of adjuvant chemotherapy and/or radiotherapy influenced the impact of survival outcomes. This might be attributed to the stage of the disease, however, as all but one advanced-stage case received chemotherapy. 

Analysis of advanced-stage disease cases that received combination therapy with chemotherapy and radiotherapy compared to those that received only radiotherapy also revealed that differences in survival intervals were non-significant, although they were shorter in patients that received only chemotherapy (RFS 35.43 (27.44, 43.43) vs. 32.12 (22.44, 41.80), *log-rank* = 0.310 and OS 52.00 (41.42, 62.56) vs. 43.91 (32.45, 55.36) *log-rank* = 0.358). The risk of relapse, which was decreased in the combined group, was also comparable (HR 0.64 (95% CI 0.25, 1.66). Similar results were obtained for the risk of death from the disease (HR 0.65, 95% CI 0.28, 1.51).

**Figure 2:**
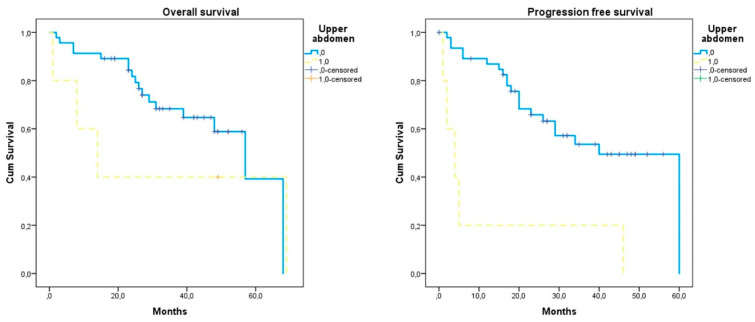
Kaplan–Meier plot of recurrence-free survival and overall survival according to the presence of upper abdominal metastases. Yellow dotted line: declined chemotherapy; blue continuous line: received chemotherapy.

Following the omission of the three cases with residual tumors that did not accomplish negative imaging results following adjuvant therapy, we observed that the recurrence-free interval of cases with incomplete tumor debulking was significantly shorter compared to that of cases with complete tumor resection (Table 2). Similarly, the risk of relapse was 4 times increased. However, neither the risk of death nor the overall survival interval was significantly differentiated from cases that had complete tumor resection. As most of these cases involved tumors that involved the upper abdominal cavity, we performed the respective analysis and observed that while the recurrence-free survival was significantly shorter (11.60 (6, 28.51) vs. 38.81 months (31.97, 45.64), *log-rank* < 0.001), the overall survival of patients did not significantly differ (32.20 (8, 62.90) vs. 48.26 months (40.58, 55.94), *log-rank* = 0.576) (Figure 2). Following inspection of the Kaplan–Meier plot, we observed, however, differences in survival curves in its intermediate section and chose to perform a Breslow analysis as well, which revealed significant differences favoring the survival of cases that did not have upper abdominal metastases (*p* = 0.040). 

**Figure 3:**
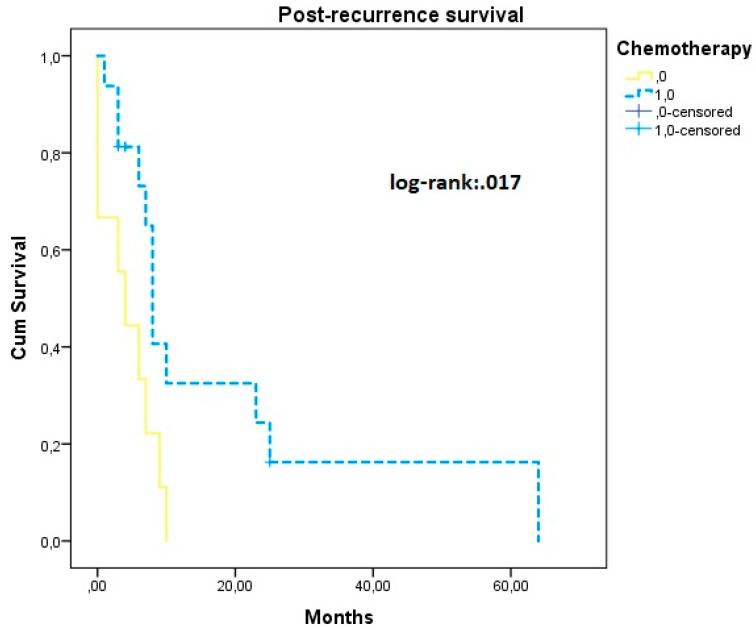
Post-recurrence survival according to the use of chemotherapy. Yellow dotted line: declined chemotherapy; blue continuous line: received chemotherapy.

Of cases that recurred, nine cases did not receive post-recurrence chemotherapy (36%) and in these patients survival analysis revealed significantly shorter post-recurrence survival rates (PRS 4.33 (1.78, 6.89) vs. 18.55 months (6.41, 30.70)) (Figure 3).

## 4. Discussion

In our study, we observed that recurrence rates of clear cell carcinoma cases accounted for approximately half of the cases in our cohort (49%). Of those cases, 81% ultimately died from the disease. Of all the risk factors that were considered as potential predictors of survival, including patient and tumor characteristics, use of adjuvant therapy and use of post-recurrence chemotherapy, only distant metastases had a significant impact on recurrence-free survival intervals, risk of recurrence and risk of death from the disease. Neither the risk of relapse nor the risk of death or the overall survival interval were significantly affected by the remaining factors that were analyzed. Upper abdominal metastases, as well as incomplete tumor resection, significantly affected recurrence-free intervals; however, the overall survival of patients was not affected. 

In accordance with the findings of previous studies, we also observed that the median age at diagnosis of CCC was older compared to that of patients with endometrioid uterine adenocarcinoma [2,10,12]. Advanced-stage disease was observed in half of cases, and lymphovascular space involvement was encountered in 71 cases, indicating the aggressiveness of the histological subtype [4]. 

Advances in adjuvant therapy have increased the survival rates of patients suffering from endometrial CCC over the last decade [13]. Nevertheless, it remains unclear why certain patients will respond to chemotherapy, whereas others will not. Survival intervals were better in patients who received combination chemotherapy and radiotherapy compared to those who received radiotherapy alone; however, the results were non-statistically significant. The use of adjuvant radiotherapy in advanced-stage disease remains debatable among several research groups [14,15,16,17]. It should be noted, however, that prospective studies are missing; hence, current recommendations [18] are based on retrospective data, as well as in prospective studies that do not specifically focus on CCC cases [19].

Following disease relapse, we observed that the use of adjuvant chemotherapy significantly increased post-recurrence survival, as expected. However, it should be noted that our findings are skewed by the fact that 4 out of 9 cases that did not receive chemotherapy were treated in a palliative manner due to low performance status and inability to tolerate chemotherapy. Nevertheless, the mean difference of 14 months is indicative of the significant and clinically meaningful effect of administering adjuvant chemotherapy. 

New data that take into consideration the molecular profile of endometrial CCC may help enhance our understanding of the actual course of the disease, as certain molecular subtypes seem to be associated with a more favorable prognosis, decreased importance of administering adjuvant chemotherapy and increased response to immune therapy [5,20,21,22]. Our study was based on a cohort of patients that was treated prior to the publication of guidelines that suggest the use of molecular profiling of endometrial cancer patients; hence, relevant studies are missing.

### 4.1. Strengths and Limitations of Our Study

The present retrospective cohort is one of the largest single-institutional published reports and is based on a consecutive patient population that is treated in an ESGO-accredited center that aims to treat patients according to the latest evidence published by international organizations. On the other hand, the retrospective design of the present study renders it susceptible to potential selection and attrition bias, thereby limiting its findings compared to those of randomized trials that should be published in this field as well. Furthermore, we encountered an issue with the information related to the percentage of patients with chemotherapy resistance and could not retrieve the relevant information, which we believe may have an impact on the course of the disease.

### 4.2. Conclusions and Implications for Future Research

The findings of our study agree with those of previously published evidence and indicate the aggressive nature of CCC in terms of patient survival. The stage of the disease at initial presentation and postoperative presence of residual tumor are the most important factors associated with survival rates, followed by the presence of upper abdominal metastases. The addition of chemotherapy does not seem to significantly influence the survival outcomes of patients with advanced-stage disease, indicating the need for further prospective research in this field. Chemotherapy should always be used following disease relapse, as it is associated with considerably increased survival rates. 

## Figures and Tables

**Table 1:** Patient, tumor and operative characteristics.

Patient, Tumor and Operative Characteristics	
Age	68 (60–76)
BMI	31 (25–35)
Stage	
I	18 (33%)
II	8 (14.5%)
III	20 (36%)
IV	9 (16.5%)
Malignant ascites	17 (31%)
Intrauterine tumor diameter (cm)	4 (3–7)
LVSI	39
Upper abdominal surgery	9
Chemotherapy	48
Radiotherapy	47
Bowel excision	5
Partial peritonectomy	8
Residual tumor	7
Declined post-recurrence chemotherapy	9

BMI: body mass index, LVSI; lymphovascular space involvement.

**Table 2:** Survival outcomes among selected subgroups of patients. Survival outcomes according to the stage of the disease, tumor diameter, presence of lymphovascular space involvement (LVSI), use of chemotherapy or radiotherapy and residual tumor following surgery.

Parameter	Progression Free Survival (95% CI)	*p* -Value	Overall Survival Months (95% CI)	*p* -Value	Hazard Ratio 95% CI PFS	*p* -Value	Hazard Ratio 95% CI OS	*p* -Value
Stage								
I	45.05 (33.59, 55.52)		56.15 (44.14, 68.16)		Ref		Ref	
II	30.04 (18.32, 42.47)	0.024	47.00 (25.71, 68.28)	0.048	2.42 (0.29, 20.23)	0.065	1.07 (0.12, 9.83)	0.079
III	36.43 (27.32, 45.55)		39.97 (31.78, 48.16)		1.16 (0.14, 9.51)		0.89 (0.11, 7.41)	
IV	15.62 (4.23, 27.02) *	0.007 *	32.56 (15.11, 40.02)		4.34 (1.52, 35.69) *	0.037	3.54 (1.81, 7.25)	0.033 *
Malignant ascites								
Absent	34.68 (27.59, 41.76)	0.248	43.82 (36.02, 51.62)	0.487	Ref	0.257	Ref	0.490
Present	30.43 (19.38, 4.48)		44.61 (33.67, 55.54)		1.58 (0.72, 3.49)		1.38 (0.55, 3.44)	
Tumor diameter	-		-		1.08 (1.02, 1.19)		1.10 (0.99, 1.21)	
LVSI								
Negative	39.40 (26.73, 52.08)	0.642	47.45 (34.60, 60.29)	0.744	Ref		Ref	
Positive	32.04 (25.59, 38.48)		46.81 (37.18, 56.44)		0.82 (0.36, 1.90)	0.641	1.15 (0.48, 2.79)	0.752
Chemotherapy								
Yes	36.22 (28.94, 32.50)	0.825	47.36 (39.22, 55.52)	0.662	Ref		Ref	
No	33.75 (18.72, 45.77)		45.56 (28.40, 57.71		1.53 (1.02, 3.30) *	0.048	1.31 (0.38, 4.57)	0.654
Radiotherapy								
Yes	39.54 (31.85, 47.25)	0.095	43.84 (34.16, 53.51)	0.637	Ref		Ref	
No	32.14 (23.54, 40.73)		44.01 (37.99, 50.04)		0.89 (0.41, 1.16)	0.090	0.54 (0.19, 1.52)	0.227
Residual tumor								
Yes	15.56 (5.70, 25.41)	<0.001	40.56 (26.63, 57.48)	0.521	Ref		Ref	
No	40.14 (32.99, 47.29) *		47.43 (38.61, 56.26)		4.04 (1.70, 9.57) *	0.004	1.38 (0.50, 3.81)	0.547

* denotes statistically significant differences.

## Data Availability

The data presented in this study are available on request from the corresponding author. The data are not publicly available due to IRB policies.
